# Surface Modification of *E. coli* Outer Membrane Vesicles with Glycosylphosphatidylinositol-Anchored Proteins: Generating Pro/Eukaryote Chimera Constructs

**DOI:** 10.3390/membranes11060428

**Published:** 2021-06-04

**Authors:** Marianne Zaruba, Lena Roschitz, Haider Sami, Manfred Ogris, Wilhelm Gerner, Christoph Metzner

**Affiliations:** 1 Institute of Virology, University of Veterinary Medicine Vienna, 1210 Vienna, Austria; marianne.zaruba@vetmeduni.ac.at (M.Z.); lena.roschitz@vetmeduni.ac.at (L.R.); 2 Laboratory of Macromolecular Cancer Therapeutics MMCT, Department of Pharmaceutical Sciences, University of Vienna, 1090 Vienna, Austria; haider.sami@univie.ac.at (H.S.); m.ogris@univie.ac.at (M.O.); 3 Institute of Immunology, University of Veterinary Medicine Vienna, 1210 Vienna, Austria; wilhelm.gerner@vetmeduni.ac.at

**Keywords:** extracellular vesicles, outer membrane vesicles, molecular painting, post-exit modification, glycosylphosphatidylinositol-anchored proteins, gene therapy, vaccines

## Abstract

Extracellular vesicles produced by different types of cells have recently attracted great attention, not only for their role in physiology and pathology, but also because of the emerging applications in gene therapy, vaccine production and diagnostics. Less well known than their eukaryotic counterpart, also bacteria produce extracellular vesicles, in the case of the Gram-negative *E. coli* the main species is termed outer membrane vesicles (OMVs). In this study, we show for the first time the functional surface modification of *E. coli* OMVs with glycosylphosphatidylinositol (GPI)-anchored protein, exploiting a process variably described as molecular painting or protein engineering in eukaryotic membranes, whereby the lipid part of the GPI anchor inserts in cell membranes. By transferring the process to bacterial vesicles, we can generate a hybrid of perfectly eukaryotic proteins (in terms of folding and post-translational modifications) on a prokaryotic platform. We could demonstrate that two different GPI proteins can be displayed on the same OMV. In addition to fluorescent marker proteins, cytokines, growth factors and antigens canb be potentially transferred, generating a versatile modular platform for a novel vaccine strategy.

## 1. Introduction

Extracellular vesicles (EV)—lipid enclosed vesicles of biological origin unable to reproduce independently—have been identified from various cellular sources. Both Gram-positive and Gram-negative bacteria produce EVs [1,2,3,4,5,6]. Cytoplasmic membrane vesicles (CMVs) are produced in Gram-positive bacteria and different species in Gram-negative, amongst them most prominently are the outer membrane vesicles (OMVs) and outer-inner membrane vesicles (O-IMVs). In addition to the roughly spherical vesicles, also tubular structures are observed (termed tube-shaped membranous structures, TSMS) [6]. They contribute to functions as diverse as quorum-sensing, horizontal gene transfer, expulsion of harmful or unwanted material, or communication in microbial consortia or biofilms, as well as mediating host–pathogen interactions [1,2,4]. Essentially, the vesicles constitute a possibility to transfer complex signaling events, or simply remove material from the bulk of the cell mass to facilitate waste disposal and toxin or antibiotic resistance. Our focus was on *E. coli* OMVs, which are produced usually in the large supernatant volumes as a side product of plasmid vector preparations. We have compared the concentration and purification methods commonly used in virology research (e.g., ultrafiltration and ultracentrifugation). This is a strategy that seems inherently reasonable, since OMVs, other EVs, and enveloped viral particles (e.g., lenti-, herpes-, influenza- or coronaviruses) have overlapping biophysical characteristics such as size and density [7]. Additionally, we tried to implement a flow cytometry approach adapted for sub-micron vesicles (also termed nanovariant flow cytometry nvFC, nanoscale flow cytometry NFC, or flow virometry) [8,9,10].

The shedding of the membrane material at the core of bacterial EV biogenesis is induced by several mechanisms, depending on the type. Outer membrane blebbing, the intercalation of hydrophobic elements into the outer membrane, shedding of LPS from the flagella, or “explosive cell lysis” (for more details see [6]) can lead to the generation of different forms of bacterial EVs. Biogenesis defines the content. All types of biological (macro-)molecules can be found in bacterial EVs. However, the subtypes may vary in a content that is more specific, i.e., cytoplasmic or inner membrane proteins are found rather scarcely in OMVs, probably indicating contamination rather than a physiological process. Similarly, RNA and chromosomal DNA are observed rarely in OMVs [6,11]. Waste disposal and communication are the two main aspects of OMV biology. Taking this into consideration, it comes as no big surprise that certain antibiotics trigger the formation of EVs to facilitate detoxification [12]. Bacterial EVs are also a crucial component of the matrix of biofilms. They serve structural functions, but also help to organize the biofilm, e.g., by increased horizontal gene transfer [13]. The targets for EVs include not only bacterial cells of the same or different species or strain [14], but also eukaryotic cells [15]. Like eukaryotic EVs [7], also their prokaryotic counterparts have a close relationship with virus particles, i.e., bacteriophages, leading both to pro- and antiviral outcomes [6,11].

Several applications for EVs in biomedicine have been discussed [16], for example, in drug delivery [17]. Due to their inherent adjuvant function, OMVs are most promising as a platform for vaccine development [18]. The platform technology has been employed for bacterial [19], protozoal [20], and viral infections such as influenza [21,22], herpes [23] or African swine fever [24]. Several issues are hindering the development of OMV-based therapeutics, amongst them are the problems in the up-scaling of preparations and toxicity issues. The strategies to circumvent these issues include bioengineering the source bacteria for enhanced vesiculation [25], manipulating the LPS structure to modulate toxicity, and decorating OMVs by different methods, either by genetic modification [25,26] or by chemical modifiers, e.g., using adaptor systems [27]. Recently, a different strategy for the surface functionalization of pro- and eukaryotic EVs has been described [28]. In such a “post-insertion” approach, first ligands are linked to polyethylene glycol-lipid micelles and subsequently incubated, originally with liposomes to allow the insertion of the micelles into the liposomal membranes. Biological membranes may be used as targets as well. The authors propose the following two different mechanisms: eukaryotic cell membranes are modified by the micelles, which give rise to EVs, and in prokaryotes, a direct insertion into the EVs is more likely [28].

We propose an alternative way to engineer OMVs by a true post-exit process. A form of surface protein engineering, commonly termed molecular painting (MP), was originally described as the spontaneous, interaction of cells with a specific type of post-translationally modified protein [29]. At the time the nature of the modification was unknown, and it was later identified as glycosylphosphatidylinositol (GPI) anchoring [30]. GPI anchors consist of a carbohydrate core structure, rich in mannose residues, and the phosphoinositol moiety that links the anchor to two to three acyl or aryl fatty acid chain residues, which are integrated into the outer leaflet of the cell membrane. The anchor is associated with the protein part via an ethanolamine linkage. The signal that targets the proteins for GPI anchoring is called a GPI signaling sequence (GSS), and it allows the anchoring of heterologous proteins when it is included in expression constructs. The technique has been tried and proven successful on both prokaryotic [31,32] and eukaryotic [33,34] cells, as well as enveloped viral particles [35,36,37,38]. A similar process has also been described for a simplified synthetic function–spacer–lipid compound, mimicking GPI anchoring [39,40]. MP has been assessed for biotechnical applications such as tumor therapy or vaccine development [35,36,37,41]. In this study, we have extended the range of the MP target membranes to bacterial EV surfaces, and thus managed to graft proteins produced in eukaryotic cells onto bacterial outer membrane vesicles, thus generating true pro/eukaryotic hybrids. The modified OMVs retain their adjuvant properties. We believe that together with the fact that more than a single protein factor may be introduced in the process, this strategy can be exploited to quickly generate vaccine preparations with high safety. The antigenic structure is of eukaryotic origin, suggesting that the glycosylation patterns and other post-translational modifications are perfectly eukaryotic (or more specifically human, if human cell lines are used for GPI-anchored protein production), indicating an advantage in the generation of immune responses in vaccination approaches. As a next step, we are assessing the use of relevant antigens, the modulation of vesiculation and adjuvant effect using different bacteria strains, and gathering further immunological data.

## 2. Materials and Methods

### 2.1. Cell Lines and Plasmids

The expression construct for GPI-anchored tdTomato was prepared in parallel to monoGGhishyg [36]. As a template for PCR (and expression construct for eukaryotic transfer capability, see Section 2.5) the commercially available expression construct pCMV-tdTomato was used (Clontech, TakaraBio). The GPI anchor was derived from the naturally glypiated protein CD55 (decay-accelerating factor, DAF). A two-step PCR mutagenesis protocol was used to combine the two parts and introduce a 6xHis tag to facilitate protein purification (first step primers: fragment 1—Tomato: T-Fw: 5′TTACTCGAGG TGAGCAAGGGCGAGGAG-3′; T-Rev: 5′GTGGTGGTGATGGTGGTGCTTGTACAGCTCGT CCATGCCGTACAG-3′; fragment 2—GSS: Bf: 5′-CACCACCATCACCACCACCCAAATA-3′; Br: 5′-CTGATGGGCCCTAAGTCAGCAA-3′). The two primary fragments were joined in the 2nd step PCR using the outer primers (T-FW and Br). The resulting fragment was used to replace the GFP/His/GSS ORF from pmonoGGhishyg [36]. The construct was transfected by calcium phosphate co-precipitation into a variety of host cell lines—CrFK (ATCC acc. no. CCL-94), HeLa (ATCC acc. no. CCL-2), HEK293T (ATCC acc. no. CRL-3216), CHO (ATCC acc. no. CCL-61)—and selected for stable expression with hygromycin B.

### 2.2. Preparation of Outer Membrane Vesicles

OMVs were prepared from *E. coli* DH5α containing a pUC19 control plasmid to facilitate selection. Small-scale cultures (<150 mL) were harvested for OMVs at an O.D. of approximately 0.8, centrifuged for 20 min at 15.000 g/4 °C in a JLA16.250 rotor on a Beckman Avanti J-25 high-performance centrifuge and frozen overnight before filtering through a 0.45 µm bottle-top filter. Large-scale preparations (150 mL to 10 L) were initially treated as small scale, but the volume was reduced by ultracentrifuging sequentially in a Type45ti rotor (2 h, 4 °C, 48.900 g) with appropriate tubes. Concentration and further purification were carried out by ultracentrifugation (with or without sucrose cushion; 2 h at 54.200 g/4 °C in an SW32ti rotor with compatible polyallomer or ultra-clear tubes) and/or ultrafiltration. For sucrose cushion, 9 mL of a 20% (*w*/*v*) sucrose solution in PBS was underlayed below 27 mL of the pre-filled OMV material. All ultracentrifugation was carried out using a Beckman Optima XL-70 ultracentrifuge. Pellets were resuspended in PBS and stored at –80 °C until further use. For ultrafiltration, spin columns from Sartorius (Vivaspin Turbo 15, molecular weight cut-off of 1000 kD) were used at 1500 to 2000 g for individual times to concentrate to a suitable volume of 90 450 µL in a Haereus Labfuge 400 R g and stored at –80 °C until further use. No final washing step was performed. Preparation/purification protocols were based on viral preparation standard protocols. Preliminary testing of a two-stage ultracentrifugation process under these conditions did not reveal a favorable influence on purity/yield, mostly due to vesicle loss.

### 2.3. Preparation of GPI-Anchored Proteins

GPI-anchored fluorophore proteins tdTomato and GFP were produced as described previously [35]. In brief, 4–6 confluent T175 flasks of GPI-AP-expressing cells were harvested by scraping after extensively washing cells with PBS. Cells were scraped into a total of 25 mL sample application buffer (50 mM TrisHCl, 50 mM NaCl, 35 mM imidazole, 1% octyl glucoside, pH 7.4). Protease-inhibitor complex (50 µL) (Sigma-Aldrich) was added. Samples were incubated for 30 min to overnight on ice before centrifugation for 30 min at 2400× *g* at 4 °C. Samples were filtered through 0.45-µm filters (Sarstedt) before application to an ÄktaPrime Plus FPLC device (GE Healthcare). Prepacked 5 mL HisTrap FF crude columns (GE HealthCare) were used. The columns were washed using washing buffer (50 mM TrisHCl, 50 mM NaCl, 25 mM imidazole, pH 7.4) and elution was achieved by using elution buffer (50 mM TrisHCl, 50 mM NaCl, 600 mM imidazole, pH 7.4). Fractions were collected during elution. Presence of GPI-anchored proteins in fractions was determined by immunoblotting and fluorometry. Positive fractions were pooled and concentrated by ultrafiltration using Vivaspin^®^ 20 ultrafiltration filter devices (Sartorius, 10 and 30 kD molecular weight cut-off, respectively) and washed twice with protein storage buffer (PSB, 50 mM Nacl, 50 mM TrisHcl pH7.4). Concentration of protein was determined using a modified Lowry assay (BioRAD Protein DC kit), according to manufacturer’s instructions. Immunoblots specific for GFP and tdTomato were performed. Fluorescence was analyzed in a Tecan plate reader (see Section 2.9). The ratio of fluorescence intensity per amount of total protein was used as an indicator of purity.

### 2.4. Molecular Painting

For painting procedures, either concentrated and purified supernatant derived from *E. coli* strain DH5α or phosphate-buffered saline (PBS) were mixed with purified GPI-anchored protein preparations in 150 µL total volume yielding a final concentration of 100–200 ng/µL. For M- and V-sample GPI-anchored proteins were replaced with PSB. After incubation for 30–180 min at 37 °C/5% CO_2_ under constant agitation, samples were diluted by addition of 36 mL of DMEM and ultracentrifuged (SW32 ti; 2 h/4 °C, 54.200 g). Samples were resuspended in 100 µL to 500 µL of PBS and stored at –80 °C for further use.

### 2.5. Pro- and Eukaryotic OMV Transfer Capability Assays

For the prokaryotic transfer test, OMV preparations derived from *E. coli* pUC19 (amp^R^) were incubated with *E. coli* strain DH5α susceptible to ß-lactam antibiotics (100 µL of overnight culture at OD 0.8). After the incubation, cells were centrifuged and plated onto ampicillin-containing agar plates [42]. Plates were then incubated at 37 °C and analyzed for colony formation.

For the eukaryotic transfer test, OMV preparations derived from the *E. coli* strains transformed with an expression plasmid coding for the protein fluorophore tdTomato (pCMVtdTomato, see Section 2.1) were incubated with different eukaryotic cell types (CrFK, NIH3T3—ATCC acc. no. CRL1658, CHO, HEK293T). Cells were centrifuged and the pellets gently mixed with the OMV preparation (20 µL) in the presence of 1 µL polybrene (stock concentration: 0.8 µg/µL) for 1 h. Then, 2 mL of DMEM/10% FCS were added, the cell suspensions were seeded onto 12-well plates and the plates were monitored for fluorescence over the following days.

### 2.6. Immunoblotting

Denatured samples were subjected to SDS-PAGE using 10% gels and a Laemmli buffer system. Proteins were blotted to PVDF membranes (GE Healthcare) under semidry conditions using a Trans-Blot^®^ SD semi-dry transfer cell (Bio-Rad Laboratories, Inc., Hercules, CA, USA) onto polyvinylidene difluoride (PVDF). After overnight blocking (4% milk powder *w*/*v*; 1% bovine serum albumin w/v in tris-buffered saline containing 0.1% Tween20), membranes were incubated with primary antibody (rabbit anti-GFP serum—Thermo Fisher cat #A-11122; goat anti-LPS/lipid A antibody—Novus NB100-64484; rabbit anti-OmpA—Abbexa abx109415; all diluted 1:1000 in tris-buffered saline containing 0.1% Tween20—TBST) and HRP-labeled secondary antibody (swine anti-rabbit, HRP, P0399 and rabbit anti-goat, HRP, P044901-2; all DAKO, Denmark; all 1:10,000 in TBST) for 1 h, respectively, including washing steps in TBST in between. Roti^®^-Lumin plus (Carl Roth GmbH) was used for generating chemiluminescence and developed using AGFA Curix 60 developer. Quantification of immunoblots was performed with ImageJ (version 1.49 t).

### 2.7. Nanoparticle Tracking Analysis

For size and concentration measurements, OMVs were diluted in PBS. Dilution factor was chosen to obtain a particle concentration of 1 × 10^8^ to 1 × 10^9^ nanoparticles/mL, which correlates to 10–100 nanoparticles per frame. For size measurement, five videos with a duration of 60 s were acquired. All samples were filtered through 0.45-µm filter prior to measurement. NTA was performed with the NanoSight NS500 (Malvern Instruments Ltd., Malvern, UK). Thereby, a laser beam was passed through a chamber containing the sample. Particles moving under Brownian motion scattered the light and single particles could be tracked by means of a microscope equipped with a camera. Background and camera levels were set manually in dependency of the particle intensity and finally, the hydrodynamic diameters and particle size distributions were analyzed by the software (NTA 3.1, Malvern Instruments Ltd., Malvern, UK) using the Stokes–Einstein equation. In order to avoid drift of the samples and to ensure reproducible results, the chamber was pre-loaded with the dilution medium (PBS) prior to and in-between each analysis until no more particles were visible.

### 2.8. Electron Microscopy

Electron microscopy was done using 50 µL of OMV suspension placed on parafilm. A formvar-laminated carbon-coated copper mesh was placed on the liquid with the coated side facing the virus suspension and incubated at room temperature for 10 min. Negative staining was performed subsequently by placing a drop of 4% phosphotungstic acid on parafilm and incubating the mesh for another 10 min at room temperature. The mesh was then allowed to dry over night and was examined the next day using a Zeiss EM900 electron microscope. Pictures were taken with ImageSP professional software.

### 2.9. Fluorometry

Samples (80–100 µL) samples were transferred to black flat-bottom 96-well plates and analyzed in a Tecan Genios plate reader (GFP: maxima@488(ex), 510 nm(em); tdTomato: maxima@554nm(ex); 581 nm(em)).

### 2.10. Flow Cytometry and Nanovariant Flow Cytometry

For assessing the immune-stimulatory potential of OMVs, *porcine* peripheral blood mononuclear cells (PBMCs) were isolated after density gradient centrifugation for 30 min at 920× *g* (Pancoll human, density: 1.077 g/mL, PAN Biotech, Aidenbach, Germany). Cells were counted on a Sysmex XP 300 hematology analyzer (Sysmex Europe GmbH, Norderstedt, Germany) prior to cryopreservation at −150 °C for future use. Details on these procedures are given in Leitner et al. (2012) [43]. After thawing, PBMCs were cultivated in U-bottom MTP (Greiner Bio-One, Kremsmünster, Austria) at a density of 2 × 10^5^ cells per well for 3 days at 37 °C and 5% CO_2_, incubated with native or modified OMVs. R848 (resiquimod, purchased from Invivogen, Toulouse, France) was used as a positive control at a final concentration of 2 µg/mL. Flow cytometry staining was caried out using the following primary antibodies: anti-CD3 (BB23-8E6, Southern Biotech, Birmingham AL, USA), anti-CD79α-PE (clone HM57, DAKO, Santa Clara, CA, USA) and anti-Ki-67-BV421 (clone B56, BD Biosciences, San Jose, CA, USA). For labelling of the CD3-specific monoclonal antibody, goat anti-mouse IgG2b-A647 (ThermoFisher) antibodies were applied as secondary reagent. During this incubation step VDeFlour 780 for live/dead discrimination (Thermo Fisher) was added according to the manufacturer’s instructions. Fixation and permeabilization for Ki-67 staining were performed using the eBioscience Foxp3 staining buffer set (Thermo Fisher) according to the manufacturer’s recommendations. Staining procedures were carried out as described previously [43]. Measurements were carried out on a BD FACSCanto II (BD Biosciences). Analysis was performed employing FlowJo software (version 10.6.2, BD Bioscience).

For nvFC we repurposed a BD FACScalibur flow cytometer for the measurement of OMVs. Samples were inactivated and filtered before measurement. The device was used on the slowest setting to reduce the likelihood of swarm effects. Additional controls include swarm effect controls (dilutions are tested to see if vesicle numbers go down according to the dilution factor), aggregation (samples containing GPI proteins only to assess degree of aggregation) and vesicle controls (samples containing only base media for assessment of non-lipid particle background). Data were analyzed using CellQuest, Flowing Software 2 and MS Excel.

## 3. Results

### 3.1. Preparation & Characterisation of E. coli OMVs

**Figure 1:**
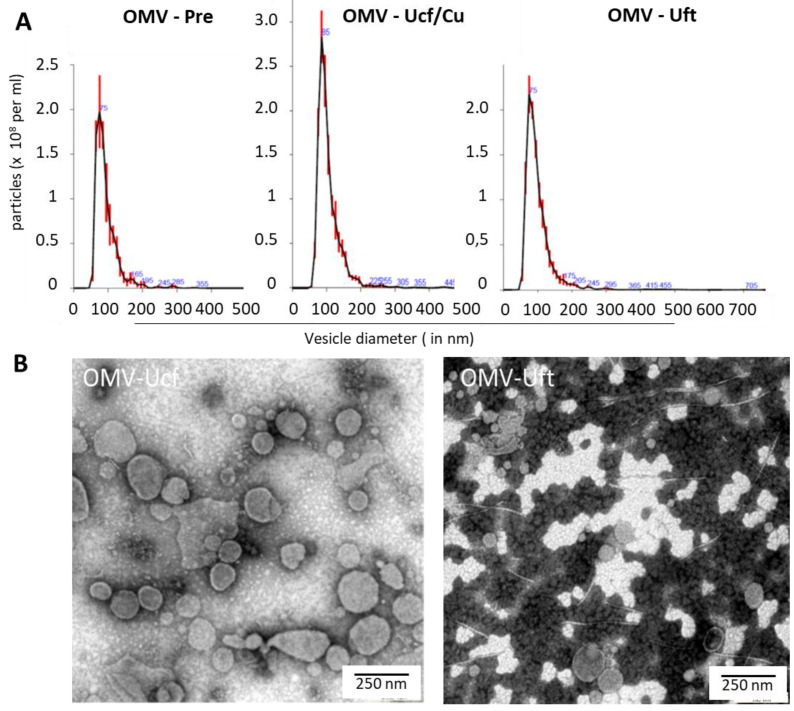
Biophysical characterization of OMVs. (**A**) The graphs show results from nanoparticle tracking analysis (NTA). The curves indicate the size distribution of particles in the samples purified by ultracentrifugation (Uct) and ultrafiltration (Uft). Material before final purification is also shown (Pre). The maximum indicates the mode of vesicle diameters. The blue numbers indicate local maxima. (**B**) The images show transmission electron micrographs of OMV preparations Light filamentous structures in the right micrograph indicate flagella debris. For quantitative analysis see Table 2.

**Figure 2:**
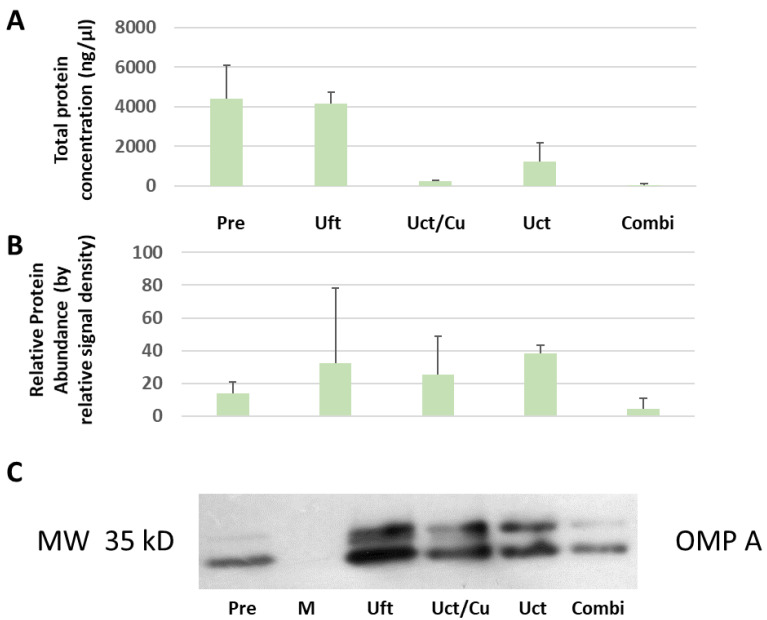
Biochemical characterization of OMVs. (**A**) The total protein concentration of various OMV preparations was measured, after using different methods for concentration (Pre, before concentration; Uft, ultrafiltration; Uct, ultracentrifugation; Cu, sucrose cushion). The strongest signal is found in the sample before concentration (see sample Pre). (**B**) Densitometry of immunoblots for ompA shows the relative abundances of OMV marker ompA. The strongest signal is found in the ultracentrifuged (see sample Uct). (**C**) Representative immunoblotting for the *E. coli* outer membrane protein A (ompA). Using this date, conclusions about the purification capability of the different methods can be made (see Table 2). All error bars indicate standard deviations. N ≥ 2.

We used the ubiquitous *E. coli* strain DH5alpha as a source for generating EV fractions from bacterial culture supernatants (BCS). In an initial characterization effort, we purified and concentrated BCS by different means, modelled on standard purification/concentration protocols used for the preparation of enveloped viral particles [7], mainly variations in ultracentrifugation and filtration protocols. All of the procedures used cultures derived from overnight cultures, grown to an O.D of approximately 0.8. The initial steps were the same for all of the protocols (centrifugation to remove bacteria and larger debris, sterile filtration to remove trace amounts of bacteria, and further debris including medium-derived aggregates (see Table 1, Figure 1 and Figure 2, labelled OMV-Pre)). Following these steps, the material was treated differently by (i) ultrafiltration (Uft), (ii) ultracentrifugation (with or without sucrose cushion, Uct and Uct/Cu, respectively) and (iii) a combination of both (Combi). The resulting samples were analyzed by electron microscopy (see Figure 1B) and NTA (see Figure 1A and compare Materials and Methods for details) to assess the morphology and size of the vesicles. Size distribution profiles, by NTA, were generated for different types of preparations (see Figure 1A). Overall, the monodispersity was good, as no pronounced secondary maxima were observed; however, a range of smaller local maxima were recorded (see Figure 1A, blue numbers).

When comparing ultracentrifugation with other preparation strategies, we found that Uct and Uft yielded on average (and in mode) larger vesicles (see Table 1), indicating that centrifugation conditions may favor the pelleting of larger vesicles or the loss of smaller (protein) aggregates.

Electron micrographs depict vesicles and different types of contamination, i.e., salt or protein aggregates, flagella debris (see Figure 1). EM pictures indicate a higher purity (less contamination) in the Uct vs. Uft samples (see Figure 1B). This is confirmed by the biochemical characteristics. The total and specific marker protein amounts were determined (see Figure 2 and Table 2).

We used the ratio of specific-to-total protein as an indicator of purity (see Table 2, relative protein abundance/protein concentration, RPA/PC). This parameter suggests that Uft/Cu yields the purest preparations.

**Figure 3:**
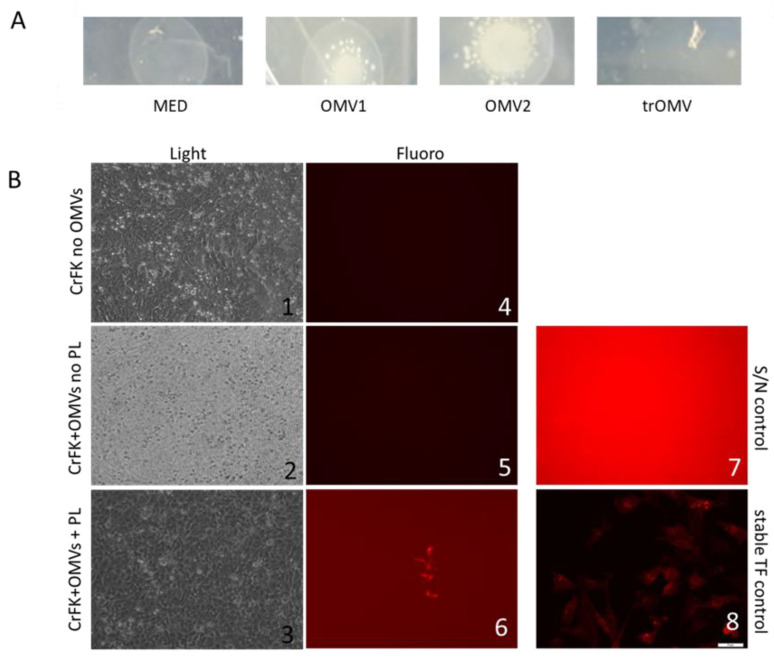
Functional characteristics—property transfer capabilities. (**A**) Prokaryotic: OMVs derived from an ampicillin-resistant *E. coli* culture were incubated with an ampicillin-sensitive *E. coli* strain. While the sensitive bacteria did not grow in the absence of OMVs (MED), bacterial growth was observed in the presence of OMVs (OMV1 and 2). When the OMVs were treated with a membrane-active substance (methyl-ß-cyclodextrin) no growth was observed (trOMVs). (**B**) OMVs were derived from an *E. coli* strain containing an expression construct for the protein fluorophore tdTomato. Different eukaryotic cell lines (CHO, CrFK, HEk293, NIH3T3) were incubated with these OMV preparations. Upon incubation, development of fluorescence was monitored. While in the absence of OMVs (see images 1 and 4) and the presence of OMVs without fluorescence plasmid cargo (see images 2 and 5) no subsequent fluorescence was observed. However, when OMVs containing the expression construct were incubated with the cells, fluorescent cells developed (see images 3 and 6). Image 7 shows an overexposed image to depict signal-to-noise ratio in samples showing no fluorescence. Image 8 shows an overlay picture of CrFK cells stably transfected with tdTomato.

We were also interested in the functional characteristics and assessed the potential of OMVs to transfer genetic or biochemical information. The OMVs were incubated with bacteria (see Figure 3A) or eukaryotic cells (see Figure 3B and Table 3), and we assessed the transfer of novel characteristics (ampicillin resistance and fluorescence activity, respectively).

In bacteria we could rescue the ampicillin-sensitive bacteria in the presence of OMVs derived from ampR bacteria (see Figure 3A). The treatment of OMVs with the membrane-active compound methyl-ß-cyclodextrin abrogated the rescue, indicating that the transferred material was associated to lipid vesicles (see Figure 3A, compare samples OMV1 and 2 with trOMV). In mammalian cells, we used OMVs derived from bacteria carrying a eukaryotic expression construct for the fluorophore tdTomato. Only in the presence of OMVs carrying the plasmid did we observe red fluorescence upon incubation (see Figure 3B and Table 3).

### 3.2. Molecular Painting of E. coli OMVs

**Figure 4:**
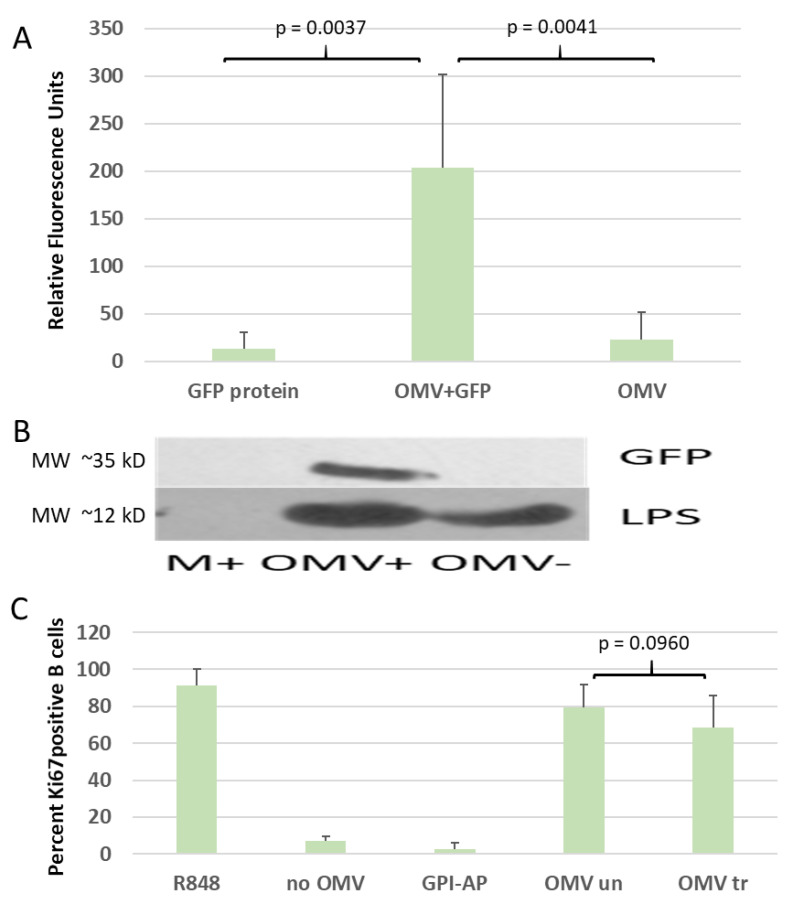
Molecular painting. GPI-anchored GFP only (GFP protein), OMVs only (OMV) and a mix of both (OMV + GFP) were incubated and OMV were separated from not associated material by ultracentrifugation. (**A**) Fluorometric data after molecular painting. Significantly higher fluorescence was observed in the OMV + GFP samples than in control samples containing GFP protein alone (GFP protein) and OMVs alone (OMV). Residual fluorescence is most likely due to residual GFP protein and vesicle autofluorescence. The *p*-values derived from *t*-test are indicated. (**B**) Immunoblotting for GFP and LPS. While a signal for GFP can only be found in the OMV + GFP samples, LPS is detected in all samples containing OMVs. (**C**) Flow cytometry analysis. *Porcine* PBMCs were incubated with OMVs to test the immune stimulatory potential of the vesicles. The B-cell proliferation marker Ki67 was used to show activation. The chemical compound R848 serves as an unspecific stimulator. In the absence of OMVs or in the presence of GPI-anchored proteins only, no significant stimulation is observed (no OMV and GPI-AP samples). OMVs show prominent B-cell stimulation, independent of whether molecular painting has been performed (compare samples OMV un and OMV tr). All error bars indicate standard deviations. N ≥ 2. The *p*-values are derived from t-test analysis.

For molecular painting (MP), OMV preparations are mixed and incubated with GPI-anchored proteins at 37 °C to allow the spontaneous insertion of GPI-AP. After incubation, the proteins not associated to the vesicles are removed by ultracentrifugation. The controls used include aggregation controls (containing no vesicles, all residual GPI-AP retained will be in the form of larger aggregates, see Figure 4) and a protein background control (no GPI-AP, to assess the presence of interfering (i.e., cross-reacting) bacterial proteins). In both of these controls, no significant fluorescence was detected after MP (see Figure 4A, samples GPI protein and OMV, respectively). Also, no GFP signal was detected in an immunoblot post-MP (see Figure 4B, compare OMV+ to OMV− and MED+). Only when both the OMVs and GPI-APs are present, is the label retained throughout the purification process (i.e., ultracentrifugation).

We were also interested to see if the inherent immuno-stimulatory activity of the OMVs was changed by the modification (see Figure 4C). For this, porcine PBMCs were stimulated in vitro for three days with OMVs. The Toll-like receptor (TLR) 7/8 agonist R848 served as a positive control since it was previously described as a potent stimulus for the proliferation of porcine B cells [44]. Total B cells were identified by gating CD79α-expressing cells (not shown), and proliferation was assessed by the proliferation marker Ki-67. The results show that the immuno-stimulatory effect between the treated and non-treated OMVs is not significantly changed (see Figure 4C, compare OMV un and OMV tr).

### 3.3. Double Painting of E. coli OMVs

**Figure 5:**
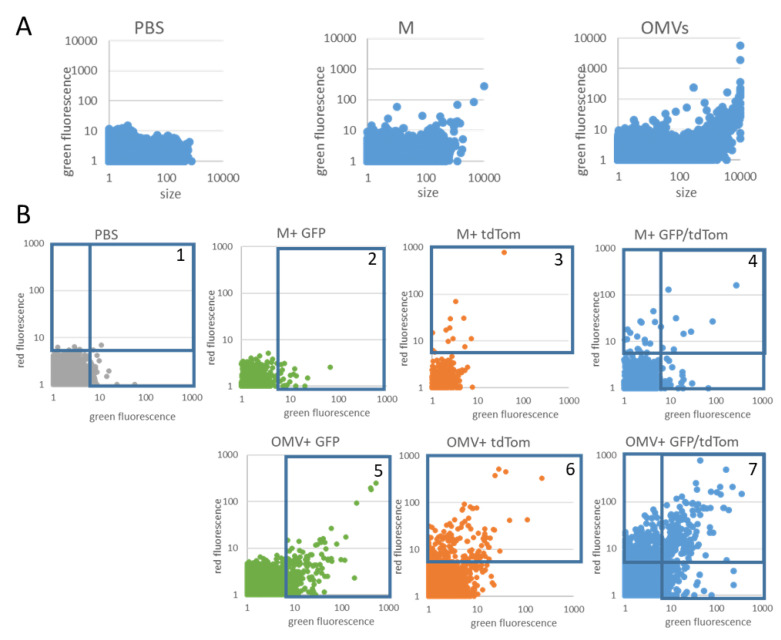
Double painting—vesicles and gates. OMV preparations modified with two different fluorophore proteins were analyzed by nanovariant flow cytometry. (**A**) Particles were measured and counted (see also Table 4) to show a significant number of events in the PBS or medium (M) controls. These are inherently not OMVs, but salt or protein aggregates or nanobubbles. A different size distribution is observed in the OMV-containing samples. (**B**) Images 1 to 7 show the distribution of gates for the different samples. For double-stained samples (4 and 7) the following two modalities are feasible: red OR green; red AND green. For quantitative data, see Figure 6.

**Figure 6:**
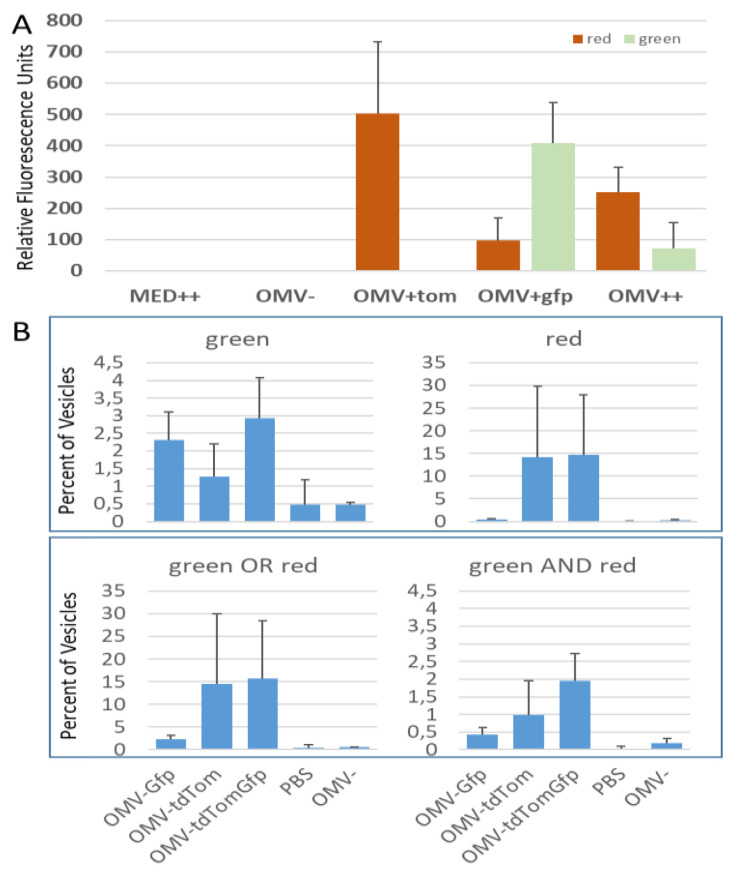
Double painting—overview of fluorescence and nanovariant flow cytometry data. (**A**) Samples after double painting were analyzed in a plate reader for fluorescence. While no fluorescence above background was detected in samples either containing no OMVs (MED++) or no GPI proteins (OMV−), red fluorescence only was observed in OMV only treated with tdTomato (OMV + tom). Significant GFP fluorescence was observed in OMV + gfp sample accompanied by a low level of red fluorescence. When both fluorophores are present (OMV++), both channels show fluorescence, but at reduced levels. (**B**) The nvFC analysis of double painting. Again, samples containing no vesicles or no OMVs show few positive events (see PBS and OMV- samples in all four graphs). OMVs carrying a green fluorescence (top left panel) are found in the samples OMV-Gfp and the double-stained OMV-tdTomGfp. GFP-positive vesicles are also observed in the OMV-tdTom sample, most likely due to the stronger fluorogenic properties of tdTomato. Red fluorescence (top right panel) is observed in the OMV-tdTom and the double-stained OMVs. The bottom left figure depicts all fluorescent vesicles (combining double- and single-labelled vesicles). The bottom right graph shows vesicles containing both, red and green fluorescence in a single vesicle. This information would not be available from fluorometry data alone. All error bars indicate standard deviations. N = 2.

For applications in drug delivery or vaccine development, the parallel incorporation of more than one factor would strongly increase the biomedical potential of the technique. For viral preparations, double painting has been described [36], however with the following serious drawback: the used method (immunoblotting) only gives averaged ensemble data (i.e., both proteins are found in the vesicle pool, no information is given about single vesicles). To rectify this situation, we established a nanovariant flow cytometry approach [8,9,10] to identify vesicles carrying both, or either/or, of the protein fluorophores (see Figure 5 and Figure 6).

First—to assess the vesicle or particle numbers in the samples—PBS, medium with GPI-AP and OMV preparations were analyzed. Many particles were found in the OMV-negative samples, indicating salt and protein aggregates and/or nanobubbles (see Figure 5 and Figure 6); however, they were mostly below a critical threshold level (see Figure 5A and Table 4).

The OMVs show a different distribution, allowing to define the gates for analysis (see Figure 6B). Quantitative analysis of the samples, using gating (as described in Figure 5B), indicated that the vesicles carrying both of the fluorophores are detectable, but at low percentages (see Figure 6B, lower right). Potential reasons for this include sensitivity issues, or a competition between the fluorophores for the available membrane space (see also Discussion). The tdTomato is a stronger fluorophore compared to the monomeric GFP variant used in this study. This probably explains why the green channel indicates a signal in the presence of tdTomato due to carry-over (see Figure 6B top left). In the red channel, only the samples containing GPI-tdTomato (OMV-tdTom; OMV-tdTomGfp) show signals (see Figure 6B, top right). Finally, also the samples showing GFP or tdTomato fluorescence are shown (see Figure 6B, bottom left).

This final set of data most closely reflects the previous immunoblotting data (ref) and the fluorometric analysis of OMV double painting (see Figure 5A). Also, in this circumstance, lower fluorescence is detected for the double-stained samples (see Figure 5A, compare OMV + tdTom; OMV + Gfp and OMV++), supporting the hypothesis that the different labels compete for membrane access.

## 4. Discussion

We have modeled the concentration and purification strategies according to procedures employed for virus preparations. The original intent was to assess how much of a contamination issue eukaryotic EVs may constitute compared to virus production levels. When applied to OMVs, the differences observed in the mean and mode of the diameter data suggests a selection for larger vesicles when using ultracentrifugation or ultrafiltration (see Figure 1 and Table 1). The appearance of a larger number of local maxima of a bigger size, in the vesicle size distribution by NTA for the ultrafiltration sample, also suggests more impurities in the Uft samples. This is confirmed by the EM micrographs. Protein aggregates and flagella debris seem to be more abundant than in the Uct samples (see Figure 1). While the use of sucrose cushions for ultracentrifugation yielded the best purity according to the specific-to-total protein ratio (see Table 2), the use of sucrose may change the surface characteristics by deposition [45]. Consequently, for physiological or functional evaluation, sucrose cushion or gradients may be less suitable, and this may apply also to other density media such as iodixanol. The standard trade-off between yield and purity applies. Both the biophysical and biochemical characterization show a better performance of Uct over Uft, at least for the conditions and products used in this study.

We also tried to assess the functional capabilities of the OMVs, more specifically, to evaluate the potential to transfer novel properties to naïve cells. In the case of the transfer to prokaryotic cells (see Figure 3A), ampicillin-sensitive bacteria were converted to resistance by incubation with OMV carrying the pUC19 plasmid (including an open reading frame for the *bla* gene). Treatment with a membrane-active substance (methyl-ß-cyclodextrin) abrogated the transfer, indicating the need for a vesicle carrier. In this setting, we did not test for the nature of the transferred factor (plasmid versus ß-lactamase protein) nor the location (i.e., whether the factors are residing on the extravesicular or luminal side of the vesicles). For the transfer to eukaryotic cells, different cell lines were incubated with OMVs containing or lacking a eukaryotic expression construct for the fluorophore. The use of a eukaryotic promotor on the expression cassette suggests (plasmid) DNA transfer, rather than protein transfer. Using protease and or DNAse/RNAse will help to elucidate such issues.

Molecular painting has originally been described under various names as a form of protein engineering for the modification of cells. We have adapted the process for enveloped viral and virus-like particles. Previously, the controls for viral MP experiments also showed GPI protein engineering in preparations derived from non-virus-producing cells, indicating the modification of other extracellular vesicles such as exosomes [37]. Bacterial vesicles have caught our attention because of their potential as vaccine platforms. Due to the severely different composition and structure of bacterial vs. eukaryotic membranes, it was not self-evident that OMVs would be suitable targets for protein engineering using GPI-anchored proteins. However, the first experiments were successful (see Figure 4A,B). It has been suggested that cholesterol may play a role in mediating MP, while others believe that membrane irregularities, independent of the type of membrane component causing them, may be responsible for MP. The fact that bacterial OMVs are amenable to MP supports the latter. The samples used in GPI protein engineering experiments contain the following two different types or control: GPI protein in the absence of lipid membranes to assess the level of aggregation of the lipophilic compound in aqueous media, and vesicles in the absence of GPI-AP to assess cross reaction or contamination.

For biomedical applications, the parallel delivery of more than one GPI-AP in one modification step is highly desirable. Along with antigens to initiate immune responses, targeting factors or further immunomodulatory elements can be introduced to the platform. Previously, we had double painted viral vector particles successfully [36]. However, data concerning the distribution of both labels on the single-particle level were missing. In this study, we have used nvFC to assess the sub-populations in the following modified samples: particles exhibiting green or red fluorescence only, vesicles containing any fluorescence, and vesicles containing both in a single entity (see Figure 5 and Figure 6). Indeed, we can identify single vesicles positive for both of the fluorophores (see Figure 6B, red AND green). As expected, the levels of double positives are significantly lower than single positives, or levels of either green or red fluorescing vesicles (see Figure 6B). Both sensitivity issues, especially concerning the weaker fluorophore (GFP), and a competition for available membrane space may lead to the low percentages observed. By choosing different fluorophores, and optimizing the vesicle-to-label ratio, the level of double positives may be increased.

We are currently identifying more relevant targets for the use in vaccine strategies, this applies to both, bacterial strains (taking into consideration vesiculation and toxicity issues) and GPI-AP, useful for manipulating immune responses (antigens, cytokines, growth factors, targeting proteins). Also, following up on the transfer capabilities of OMVs targeting eukaryotic cells should help to assess the use of OMVs also as a DNA/RNA delivery tool.

## 5. Conclusions

We have prepared and characterized OMV from *E. coli* cultures in various ways, identifying ultracentrifugation (with or without sucrose cushion) as the preferable option for OMV production. From our data, we conclude that the direct surface modification of outer membrane vesicles is feasible, allowing the deposition of eukaryotic proteins on a prokaryotic background. The immune stimulatory properties of the OMVs (e.g., B-cell stimulation) are not significantly changed. Parallel deposition of two different proteins is possible and we could demonstrate the presence of both fluorophores on the same vesicle with nvFC.

## Figures and Tables

**Table 1:** Nanoparticle tracking analysis—diameter and concentration.

Sample	Diameter Av. (in nm)	Diameter Mode (in nm)	Concentration (Particles/mL)
OMV Pre	93.5+/−2.0	73.9+/−2.6	9.25 × 10^12^
OMV Ucf	104.8+/−1.0	88.0+/−1.8	6.35 × 10^13^
OMV Uft	101.7+/−2.7	80.6+/−2.0	1.65 × 10^14^

Av., average; Pre, before concentration; Ucf, ultracentrifugation; Uft, ultrafiltration.

**Table 2:** Estimating purity of OMV preparations.

	Protein Concentration (PC, ng/µL)	Relative Protein Abundance (RPA, Signal Density)	RPA/PC
Pre	4418	14	3.2
Uft	4133.5	32.5	7.9
Uct/Cu	233	25.5	109.4
Uct	1246.5	38.5	30.9
Combi	48	4.5	93.8

Pre, before concentration; Uft, ultrafiltration; Uct, ultracentrifugation; Cu, sucrose cushion; Combi is Uft and Uct.

**Table 3:** OMV-mediated fluorescence transfer.

	CrFK	HEK293T	NIH3T3	CHO
No OMVs	−	−	−	−
OMVs tdTom	++	+	−	−
Stable transfected	++++	++++	++++	++++

(−) to (++++) no to strong fluorescence observed.

**Table 4:** Vesicle overview.

	PBS	M	OMV
**All events**	204,901	103,051	97,200
Vesicles *	492	484	9983
**% of all**	0.2	0.5	10.3

* Threshold about 30, numbers derived from double-labelled samples.
